# Intersecting Epidemics: Examining the Impact of Internalized Homophobia and Depression Symptoms on HIV Testing Through a Suicide Syndemic Among Young Black Men Who Have Sex with Men

**DOI:** 10.1007/s40615-024-02225-4

**Published:** 2024-10-30

**Authors:** Donte T. Boyd, Omar Martinez, Tural Mammadli, Osman Wumpini Shamrock, Gamji Rabiu Abu-Ba’are, Typhanye V. Dyer

**Affiliations:** 1 College of Social Work, The Ohio State University, Columbus, OH USA; 2 Center for Interdisciplinary Research On AIDS (CIRA), Yale University, New Haven, CT USA; 3 Center for AIDS Prevention Studies (CAPS), UCSF Medical Center at Mission Bay, San Francisco, CA USA; 4 College of Medicine, University of Central Florida, Orlando, FL USA; 5 School of Social Work, University of Maryland, Baltimore, MD USA; 6 School of Nursing, University of Rochester, Rochester, NY USA; 7 School of Public Health, Departments of Epidemiology and Biostatistics, University of Maryland, College Park, MD USA

**Keywords:** Mental Health, HIV testing, Young Black MSM, Suicide Syndemic

## Abstract

Young Black men who have sex with men (BMSM) in the USA face disproportionate rates of HIV incidence. Mental health vulnerabilities, including depression, anxiety, substance use, and trauma, further exacerbate the HIV epidemic among this population. Internalized homophobia, discrimination, and depression contribute to elevated rates of suicidal behavior among young BMSM, which in turn may influence engagement in HIV prevention behaviors, such as HIV testing. However, limited research has examined the interplay among suicidal behaviors, internalized homophobia, depression, and HIV testing among young BMSM. This study utilized syndemic theory to explore the relationships among these factors in a sample of 400 young BMSM ages 18–29. Results indicate alarming rates of suicidal behavior among young BMSM, with significant associations among internalized homophobia, depression symptoms, suicidal behavior, and HIV testing. The findings underscore the urgent need for targeted mental health interventions and HIV prevention services tailored to address the unique challenges faced by young BMSM. Comprehensive, multi-level, community-centered interventions are essential to address the syndemics affecting young BMSM, promoting holistic health and well-being while improving outcomes across the HIV prevention continuum.

## Background

Black men who have sex with men (BMSM) in the USA are overrepresented in HIV incidence [1–4]. The current lifetime risk of acquiring HIV among BMSM is 50%, with some estimates suggesting that upward of 60% of BMSM could acquire HIV by the time they reach 40 years old [1]. However, in the USA, internalized homophobia, discrimination, depression, and other factors are likely to contribute to reporting bias and underestimates by men who have sex with men (MSM) [5, 6].

In the past 40 years, increasing attention has been focused on the multidimensional mental health vulnerabilities of men who have sex with men, including issues such as depression, anxiety, substance use, and trauma [6]. For instance, in a survey of 829 BMSM from 41 states across the USA, rates of depression among participants were found to be as high as 33%, nearly five times higher than the national rate for all adults [7]. BMSM experience the adverse impacts of both racism and homophobia, resulting in greater disparities in depression and other mental health outcomes compared to other racial and ethnic minority groups [7–9]. Depression within the BMSM community is linked to an increased risk of HIV transmission [7]. Addressing these overlapping challenges requires tailored interventions that account for the unique experiences of MSM, providing comprehensive mental health support, and reducing barriers to care such as racism, homophobia, and lack of access to culturally competent providers.

In addition to depression, emerging research has suggested that suicide rates for LGBTQI + individuals, including BMSM, are alarming. Young men ages 18 to 25 face elevated rates of suicidal ideation, with 18.4% of gay men and 22.2% of bisexual men experiencing it [10–12], in contrast to 6.7% of heterosexual men [10–12]. Similarly, 7.4% of gay and bisexual men in this age group have planned suicide attempts, compared to 1.9% of heterosexual men [10–12]. Additionally, 3.3% of gay and bisexual young men have attempted suicide, while only 1.1% of heterosexual young men have done so [10–12]. In a recent study among a national sample of 400 BMSM ages 18 to 29, 33% of the sample reported planning to die by suicide and 27% attempted suicide [10]. Addressing mental health and suicide among BMSM may increase HIV prevention behaviors, including HIV testing.

HIV testing plays a vital role in both national and local strategies aimed at stemming the HIV epidemic. It serves as the entry point to the HIV prevention continuum, allowing individuals to become aware of their status and access necessary care, including pre-exposure prophylaxis (PrEP) and antiretroviral therapy (ART) [13]. Though rates of HIV testing have increased over time, there remains a notable number of MSM who have never been tested for HIV, especially those under age 25 [13]. Research into barriers to HIV testing has examined factors such as stigma, discrimination, poverty, and substance use, but there is a dearth of literature focused on how internalized homophobia [13] and depression symptoms influence HIV testing, and to our knowledge, there is a dearth of literature that examines how suicidal behaviors influence HIV testing among BMSM.

## Introduction

### Internalized Homophobia and HIV Testing

As a critical component of HIV prevention as well as a connection to HIV care, understanding factors that affect HIV testing or lack thereof is an important part of reducing HIV-related disparities among BMSM [14]. Literature examining HIV vulnerability among MSM has found societal factors such as stigma to be among the drivers of the disproportionate HIV burden faced by MSM [15–19]. BMSM experience high levels of stigmatizing events, such as discrimination related to their sexual orientation [18, 19]. Such stigmatizing experiences have been linked to a higher likelihood of engagement in behaviors contributing to HIV vulnerability, including engagement in unprotected anal sex or sex under the influence of substances, use of preexposure or postexposure prophylaxis, and HIV testing [20–24].

One pathway connecting homophobic stigma and HIV burden is through the internalization of stigma experiences [25]. As a result of persistent stigmatizing experiences, MSM may come to internalize negative attitudes and beliefs associated with their sexual minority identity [25]. Previous work suggests higher internalized homophobia may cause reduced engagement in HIV prevention behaviors, including HIV testing, among MSM [26–29]. In a recent study of 907 Korean men who identify as gay or bisexual, lower internalized homophobia predicted higher HIV testing prevalence [27]. Although literature examining the relationship between internalized homophobia and HIV testing among BMSM is limited, it similarly predicts lower testing among those with higher internalized homophobia [28, 29]. For instance, a study of 4174 BMSM recruited from Black Pride events in six US cities (Atlanta, Detroit, Houston, Memphis, Philadelphia, and Washington, DC) between 2014 and 2017 found that BMSM who reported higher levels of internalized homophobia were 1.4 times more likely to have never engaged in HIV testing relative to their counterparts with lower internalized homophobia [13].

### Depression and HIV Testing

Alongside internalized stigma, mental well-being plays an important role in HIV vulnerability among MSM. Literature indicates that the presence of mental health conditions such as depression is linked to higher odds of HIV acquisition [30–32], and the prevalence of HIV is higher among those with mental illness, including depression [30, 33, 34]. It is suggested that people suffering from depression may be more likely to engage in sexual behaviors considered higher risk for HIV acquisition, such as the use of substances prior to sex and unprotected sex [30, 35]. People suffering from depression may also exhibit lower preexposure prophylaxis adherence and potentially lower HIV testing [36–39].

Evidence on HIV testing odds is conflicting, as several studies, including a 2009 systematic review [38], suggested lower HIV testing prevalence among those experiencing severe mental illness [36]. Yet there is work that suggests otherwise, demonstrating higher levels of HIV testing among those living with depression or other mental illnesses [13, 37, 40]. Similarly, limited literature suggests a complicated relationship between depression and HIV testing among BMSM. In the aforementioned study of 4174 BMSM recruited across six cities, Matthews et al. [13] found that BMSM with depressive symptoms were approximately 1.5 times more likely to have never tested for HIV than to have tested at least once in their lifetime. Likewise, another study of 1553 Black sexual minority men elucidated a lower likelihood of having been tested for HIV among those with depressive symptoms [41]. These findings are in contrast with those reported by Chandler et al. [42], who did not find differences in past-6-month testing frequency based on the presence of moderate to severe depression symptoms, independently, in a sample of 3,294 BMSM living in the United States. They did find, however, that synergy between polydrug use and depression and problematic drinking and depression resulted in a lower likelihood of HIV testing in the past six months [42]. This is not an isolated finding, as studies with differing samples have demonstrated that lower HIV testing prevalence among those with mental health concerns is actualized in the presence of other syndemic factors [43, 44].

### Suicidality

Sexual minority adults consistently experience higher rates of suicide attempts compared to their heterosexual counterparts, highlighting ongoing disparities in mental health outcomes [45–47]. Generally, higher rates of suicide are more commonly observed in White populations as compared to Black and Hispanic populations [48, 49]. However, bourgeoning research suggested that BMSM are at severe risk for suicide [10, 50, 51]. In one recent study that used a national sample of 497 Black and 1536 White sexual minority males (ages 16 to 25), results indicated that among Black participants, structural racism and anti-LGBTQI + policies were significantly correlated with increased levels of depressive symptoms, heavy drinking, perceived burdensomeness, thwarted belongingness, self-harm behaviors, and suicide attempts [52]. In another online national study among 400 BMSM ages 18 to 29, results indicated that internalized homophobia and depression symptoms increased suicidal attempts [50]. However, the literature on suicidality among young BMSM has been significantly understudied, and no studies have examined the potential link between suicidality and HIV prevention behaviors in this population.

### Syndemic Theory

Syndemic theory posits that epidemics of multiple physical, psychological, social, and structural factors co-occur among disadvantaged groups due to adverse social conditions [53]. Syndemic frameworks have been utilized to explain elevated HIV risk in sexual and gender minority populations (SGM) [53–59] but have only recently been applied to examine the impact of a suicide syndemic on engagement in the continuum of care for young BMSM of unknown HIV status [60–62]. A syndemic is defined as the co-occurrence of two or more conditions that interact synergistically to increase the burden of disease outcomes [63].

While extant literature highlights the health effects associated with each of the individual conditions that characterize a suicide syndemic (planning, ideation, and attempts), which are well documented and salient for population health [64, 65], the health effects of a potential suicide syndemic on HIV testing are scant. Suicidal planning, ideation, and attempts, which are seemingly sequential in nature, theoretically represent a syndemic, wherein exposure to one factor reinforces the co-occurrence of the other factors, producing synergy, reciprocity, and multi-directionality among the factors [66–68].

These factors are known to independently influence HIV testing [69, 70]; however, the synergistic impact of all three suicide factors on HIV testing is less understood. Moreover, mediating pathways that are indirectly affected by the suicide syndemic and their impact on HIV testing have yet to be tested. Furthermore, it is unclear whether such syndemic manifestations and their association with HIV testing are more salient in young BMSM at increased risk of acquiring HIV compared to young BMSM whose experiences do not form a syndemic.

Internalized homophobia and depressive symptomatology are known factors that impact engagement in care for people living with HIV [71, 72], the first step of which is to know your status by getting tested for HIV. Suicidal planning, ideation, and attempts may form a suicide syndemic, characterized by these three factors not only co-occurring but also mutually reinforcing one another.

### Current Study

Synergistic and mutually reinforcing mental health and the HIV epidemic are concentrated in the communities of young BMSM and derive from social and structural inequities that exacerbate illness and disease in this population. The current study is guided by syndemic theory to elucidate ways in which mental health and suicidal behaviors influence HIV testing among young BMSM. Suicidal behaviors may be potentially important factors, along with IHP and depression symptoms that co-occur, reinforce one another, and may ultimately contribute to HIV prevention behaviors. Young BMSM who experience depression symptoms or suicidal thoughts and behaviors or who wrestle with internalized homophobia may be less likely to engage in HIV testing. We hypothesize that (a) internalized homophobia will be positively associated with depression symptoms, (b) depression symptoms will be positively associated with suicidal factors, (c) suicidal ideation and attempts will be negatively associated with HIV testing, (d) internalized homophobia will be indirectly associated with HIV testing, and (e) depression symptoms will be indirectly associated with HIV testing.

## Methods

### Study Procedures and Recruitment

The survey was programmed with Qualtrics software for different sampling sites. An anonymous link was generated and included on a recruitment flyer, which was then distributed via social media sites (Facebook and Twitter) and provided to community-based organizations and Amazon Mechanical Turk (M-Turk) [10, 50, 51]. The principal investigator and research assistants distributed the survey via social media every morning at 8 a.m. Eastern time.

Amazon M-Turk offers a cost-effective and speedy recruitment method for research across various fields, including public health [10, 50, 51]. To access and participate in the survey, M-Turk registrants needed to have a 95% or higher approval rating from previous surveys, be 18 years or older, and reside in the USA, as verified during their initial M-Turk registration [10, 50, 51]. Furthermore, individuals logging into the M-Turk platform during the survey week were informed of the opportunity to take a survey focused on HIV and assets for BMSM. They were told that the survey would require approximately 20 min and would be available daily at 8 a.m. Eastern Standard Time. Participants were instructed to complete the survey in one session, and they received a US$1 compensation along with other incentives from M-Turk [10, 50, 51].

The research team collaborated with community-based organizations, providing them with the survey flyer. Community health workers, in turn, shared this flyer with their eligible clients, including an anonymous survey link. Recruitment occurred between 1 December 2021 and 31 January 2022. Participants who completed the 20-min survey and provided their e-mail received a $35 Amazon gift card. For data quality and to prevent bots, our survey employed Qualtrics survey protection. We also verified respondents’ IP addresses to confirm their residence in the USA, thus ensuring data integrity by preventing duplicate responses to eligibility and survey questions. Furthermore, we used speeding checks to exclude from the final sample participants with survey durations less than one-third of the median survey duration. Qualtrics survey protection provided tools to prevent fraudulent submissions, including a ballot box-stuffing prevention tool that places a browser cookie after a response; reCAPTCHA scores, which required respondents to identify items in pictures or replicate letters; and bot detection through a reCAPTCHA score indicating the likelihood of a respondent being a bot.

### Participants

Consistent inclusion/exclusion criteria were applied across all sampling sites. Eligible participants for the study were those who self-identified as Black or African American, were ages 18 to 29, lived in the USA, were assigned male at birth, were proficient in English, currently identified as a man, and reported having anal intercourse with another male in the previous 12 months. Respondents who did not meet these criteria were promptly removed from the survey. We enforced a forced response option in Qualtrics to ensure every participant answered each question.

After clicking the survey link, participants received information about the study and were requested to complete a screening tool to determine their eligibility. The verified participants answered questions on demographics, developmental assets, mental health, and other protective factors. The study received approval from the Ohio State University Institutional Review Board (IRB No. 2021E1175).

The final sample comprised 400 BMSM ages 18 to 29 (M = 23.46; SD = 2.59). The majority of the participants (*n* = 200) were recruited from M-Turk, followed by community-based organizations (*n* = 100) and social media sites (*n* = 100). The majority of the sample identified as Black American or African American (75%), followed by Caribbean (10%) and Afro-Latino (10%), and 5% self-identified as continental African. A total of 28% of the sample never attended high school, while 29% completed college or were postgrads. The average household income ranged from < $20,000 to $150,000, the average being $57,499. The majority of the sample (95%) reported being assigned male at birth and 5% as female. All the participants (100%) self-reported having sex with men within the previous year; 45% reported being gay, 35% straight or heterosexual, 10% bisexual, 5% questioning, and 5% other.

### Measures

#### Dependent Variable: HIV Testing

Participants’ responses to the following defined HIV testing: “Have you, yourself, ever been tested for HIV in the last 12 months?” The responses were coded using a dichotomous response (0 = *no*, 1 = *yes*).

#### Mediator: Suicide Syndemic Risk Factor

The suicide syndemic factor included suicide planning, ideation, and attempts. We assessed suicide attempts using a single item that asked respondents to indicate whether they had attempted to end their life within the previous 12 months. Response categories were 1 = *yes* and 0 = *no* [39]. Suicide planning was assessed with a single item that asked participants whether they had made a plan to end their life within the previous 12 months. Response categories were 1 = *yes* and 0 = *no* [39]. We assessed suicide attempts using a single item that asked respondents to indicate whether they had attempted to end their life within the previous 12 months. Response categories were 1 = *yes* and 0 = *no* [39]. Suicide ideation was measured using a single item that asked respondents to indicate whether they considered ending their life in the previous 12 months. Response categories were 1 = *yes*, 0 = *no*. We created a latent variable with these three observed variables (suicide attempts, suicide planning, and suicide ideation). This latent construct was tested for model fit using the chi-square, root square mean error, Tucker–Lewis index, and comparative fit index**.**

#### Independent Variables

##### Depression Symptoms

We used the Center for Epidemiological Studies Depression Scale (CESD-10) to measure depression symptoms [43]. The CESD-10 assesses depressive symptoms experienced in the past week. Prior research has validated the measure among clinically depressed populations, the general population, and sexual minorities of color [7]. Sample items included “How many times in the past week did you feel as good as other people?” and “How many times in the past week did you have trouble keeping your mind on task?” Response options range from zero (*Rarely or never*) to 3 (*Most or all of the time*). The CESD-10 scores range from zero to 30, with higher scores indicating more depressive symptoms (Cronbach’s *α* = 0.81). Individuals with scores above 20 were classified as having moderate to severe depression symptoms [44].

##### Internalized Homophobia

Internalized homophobia was measured using nine items on a 5-point Likert scale ranging from 1 (*Strongly disagree*) to 5 (*Strongly agree*). This scale assessed the extent to which lesbian, gay, and bisexual individuals reject their sexual orientation, are uneasy about their same-sex desires, and seek to avoid same-sex attractions and sexual feelings. Sample items include “I often feel it best to avoid personal or social involvement with other gay/bisexual men” and “I feel alienated from myself because of being gay/bisexual.” For the current study, responses to the nine items were summed to yield a total score for internalized homophobia (M = 25.03, SD = 11.23; Cronbach’s *α* = 0.96).

### Data Analysis

Preliminary data analyses included the examination of normality, alpha-level (*α*) reliabilities, and descriptive statistics. We calculated descriptive statistics to convey the distribution of these constructs within the sample. Table 1 presents demographic statistics of categorical key study variables, and Table 2 presents continuous key variables (*N* = 400). Table 3 presents bivariate correlations of study variables.

**Table 1:** Demographic categorical study variables (*N* = 400)

Variable	Frequency	Percent
Suicide ideation		
Yes	128	38
No	206	62
Planning to die by suicide		
Yes	130	34
No	220	66
Suicide attempts		
Yes	98	28
No	252	72
HIV testing		
Yes	180	45
No	220	55

**Table 2:** Demographic continuous study variable (*N* = 400)

Variable	Mean	Standard deviation	Range
Internalized homophobia	3.0	1.24	1–5
Depression symptoms	14.46	5.97	0–30

**Table 3:** Bivariate correlations of key study variables (*N* = 400)

Suicide attempts	1				
Suicide planning	0.62*	1			
Suicide ideation	0.67*	0.74*	1		
Depression symptoms	0.40*	0.45*	0.06	1	
Internalized homophobia	0.41*	0.50*	0.06	0.62*	1

Note: *p < 0.05

**Fig. 1:**
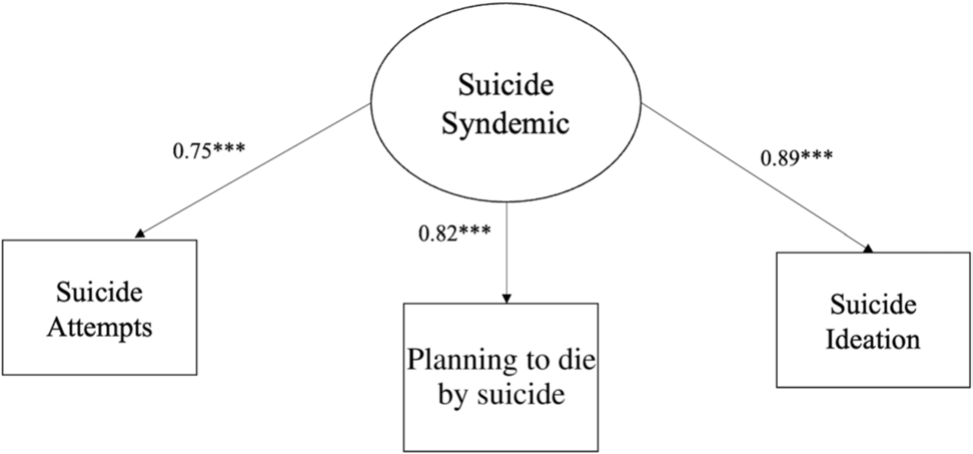
Measurement model of the suicide syndemic. Standardized betas are reported, with all variables significant at ***p < .001

**Fig. 2:**
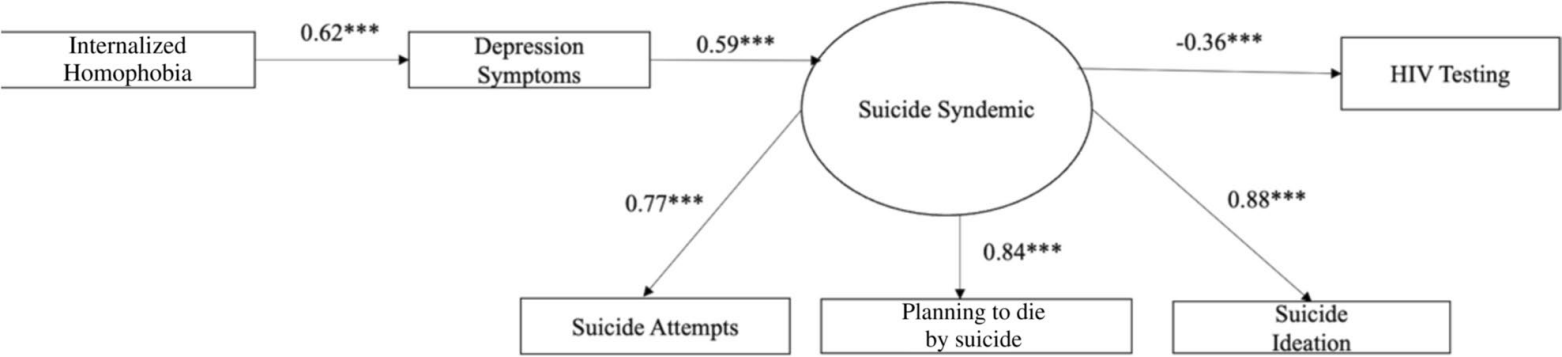
Path model testing direct and indirect effects of internalized homophobia on HIV testing via depression symptoms and the suicide syndemic. Standardized betas are reported, with all variables significant at ***p < .001

Our first step in the main analysis was to conduct a confirmatory factor analysis to test a measurement model of suicide syndemic (Fig. 1). The model fit was assessed using the model Chi-square, the root mean square error of approximation (RMSEA), the comparative fit index, and the Tucker–Lewis index. Once an adequate fit was determined, we performed structural equation modeling using M-Plus version 8.3. We investigated whether internalized homophobia and depression symptoms were associated with HIV testing through a suicide syndemic (Fig. 2). The mean-and-variance-adjusted weighted least squares estimator was used, which is preferred when the dependent variables are categorical and when data are not normally distributed. Standardized beta coefficients and *p* values were included (Table 4).

**Table 4:** Direct effects on HIV testing via internalized homophobia, depression symptoms, and the suicide syndemic among Black MSM aged 18 to 29 (*N* = 400)

	Unstandardized beta (B)	Standardized beta (β)	95% confidence interval
Depression symptoms			
Internalized homophobia→	3.04	0.62***	0.57, 0.70
Suicide syndemic			
Depression symptoms→	0.03	0.59*	0.50, 0.67
Measurement model			
Suicide attempts	1 (constrained)	–	–
Suicide syndemic			
Suicide planning→	1.16	0.84*	0.79, 0.89
Suicide syndemic			
Suicide ideation→	1.26	0.87*	0.83, 0.92
HIV testing	–	–	–
Suicide syndemic→	− 0.49	− 0.36*	− 0.66, − 0.46

Note: *p < .05; ***p < 0.001

## Results

In total, 28% of BMSM reported that they had attempted suicide, 34% of the sample reported that they had planned for suicide, and 38% self-reported that they thought about suicide (Table 1). BMSM reported moderate forms of internalized homophobia (M = 3.0; SD = 1.24). The mean for depression symptoms among this population was 14.46 (SD = 5.97), which means the young in this sample are depressed (Table 2). Correlation results (Table 3) showed that suicide planning was positively associated with suicide attempts (*r* = 0.62, *p* < 0.001). Suicide ideation was positively associated with suicide attempts (*r* = 0.67, *p* < 0.001) and suicide planning (*r* = 0.74, *p* < 0.001). Depression symptoms were positively associated with suicide attempts (*r* = 0.40, *p* < 0.001) and suicide ideation (*r* = 0.45, *p* < 0.001). Internalize homophobia was correlated with suicide attempts (*r* = 0.41, *p* < 0.001), suicide planning (*r* = 0.50, *p* < 0.001), and depression symptoms (*r* = 0.62, *p* < 0.001).

### Measurement Model

For the mediator, a latent factor was formed using items from three separate constructs of suicidal behavior. The latent factor was formed to create a suicide syndemic. Factor loadings on the suicide syndemic ranged from 0.75 to 0.89. The suicide syndemic provided a good model fit: *χ*^2^(3) = 93.87, *p* = 0.51, RMSEA = 0.02, comparative fit index = 0.99, Tucker–Lewis index = 0.99 (Fig. 1).

### Structure Equation Model

The structural equation modeling results on HIV testing, including standardized betas and *p*-values, are displayed in Fig. 2. The model examined the direct and indirect associations between internalized homophobia, depression symptoms, suicide syndemic, and HIV testing. The hypothesized model also demonstrated a good fit for the study data (Table 4). Suicide syndemic explained 70% of the variance in HIV testing. Our results indicated that internalized homophobia was directly and positively associated with depression symptoms (*β* = 0.63, *p* < 0.001). Depression symptoms were direct and positively associated with suicide syndemic (*β* = 0.58, *p* < 0.001). Lastly, suicide syndemic was directly and negatively associated with HIV testing (*β* =  − 0.36, *p* < 0.001). Internalized homophobia was indirectly and negatively associated with HIV testing (*β* =  − 0.05, *p* = 0.01). Depression symptoms were also indirectly and negatively associated with HIV testing (*β* =  − 0.02, *p* < 0.001) (Table 5).

**Table 5:** Indirect effects on HIV testing via internalized homophobia, depression symptoms, and the suicide syndemic among Black MSM aged 18 to 29 (*N* = 400)

	Unstandardized beta (B)	Standardized error	95% confidence interval
HIV testing			
Internalized homophobia→	− 0.05	0.01	− 0.07, − 0.03
Depression symptoms→	− 0.02*	0.00	− 0.02, − 0.01
Suicide syndemic			
Internalized homophobia→	0.10*	0.02	0.07, 0.12
Suicide attempts			
Internalized homophobia→	0.10	0.01	0.08, 0.14
Depression symptoms→	0.03*	0.00	0.02, 0.04
Suicide planning			
Internalized homophobia→	0.11	0.01	0.09, 0.14
Depression symptoms→	0.04*	0.00	0.03, 0.04
Suicide ideation			
Internalized homophobia→	0.12	0.01	0.09, 0.15
Depression symptoms→	0.04*	0.00	0.03, 0.05

Note: *p < 0.05

## Discussion

This is one of the first studies to investigate the effect of depression symptoms, internalized homophobia, and a suicide syndemic on HIV testing among young BMSM. Our results indicated that depression symptoms and internalized homophobia exacerbate suicide among this population. In addition, the suicide syndemic lowered HIV testing, which can potentially prolong the HIV epidemic. We propose prioritizing the promotion of mental health and suicide prevention among young BMSM as a public health strategy that could help to end the HIV epidemic. Improving mental health and reducing suicide itself is an underrecognized public health priority for this population in the USA.

The findings of this study reveal concerning and alarming rates of suicidal behavior among young BMSM, with a significant proportion reporting suicide attempts (25%), planning to die by suicide (28%), and suicidal ideation (31%). These rates underscore the urgent need for targeted mental health interventions and support services tailored to address the unique challenges faced by young BMSM, including racism and discrimination, stigma, lack of mental health resources, and safety net clinics [10, 50–52]. These findings are consistent with the existing literature on suicide disparities among BMSM [10, 73–75]. Addressing suicide among young Black men requires a multifaceted approach that considers the intersections of race, sexuality, and mental health, which can help them achieve optimal health.

Prevalence estimates of internalized homophobia and depression symptoms among the study participants highlight the intersecting psychosocial stressors experienced by young BMSM, which may contribute to their heightened vulnerability to suicidal behavior. This is consistent with research that reported that depression severity and internalized homophobia directly and indirectly increased suicide attempts [76, 77]. Internalized homophobia, in particular, emerges as a significant correlate of suicidal behavior, suggesting the detrimental impact of societal stigma and discrimination on the mental health and well-being of BMSM individuals [77–80].

When developing and designing interventions and prevention programs for young Black BMSM, it is critical to adopt culturally competent frameworks rooted in an Afrocentric paradigm. Afrocentric priorities emphasize key principles and practices that celebrate African cultural norms, values, and perspectives [81, 82]. This approach aims to affirm and honor African identities and ways of knowing, often in response to historical marginalization and the dominance of Eurocentric viewpoints [81, 82]. By integrating these elements, we can effectively address the unique experiences and challenges faced by young BMSM, particularly concerning internalized homophobia and depression [83–86]. For example, cultural affirmation and pride—highlighting the richness of African cultures, languages, traditions, and histories—can be woven into programs that promote pride in African heritage and foster positive narratives surrounding Black identity and LGBTQ + community membership.

Moreover, incorporating social justice and liberation into mental health programs is essential for advancing equity and justice within African American communities. This approach involves actively teaching young men to help dismantle systemic racism, discrimination, and all forms of social injustice that impede the well-being and progress of these communities. By teaching them to advocate for LGBTQ + rights and racial equity, we can effectively tackle external sources of stress and discrimination. Additionally, it is vital to educate young Black MSM about systemic issues and empower them to become agents of change within their communities. By integrating these Afrocentric priorities [81, 82], mental health and HIV interventions can become more culturally relevant and supportive, helping to reduce internalized homophobia and depression among young Black men and fostering a greater sense of belonging and well-being.

To our knowledge, this is the first study to create and utilize a suicide syndemic. The suicide syndemic framework, comprising items related to suicide attempts, planning, and ideation, provides a comprehensive approach to understanding the complex interplay of suicidal behaviors within this population. The robustness of the suicide syndemic model underscores the interconnectedness of various suicidal indicators and their collective influence on mental health outcomes among young BMSM. By addressing the interconnected factors contributing to the suicide syndemic among young BMSM and implementing a coordinated and collaborative approach that centers on their unique needs and experiences, we can work toward reducing suicide rates and promoting the overall well-being of this population.

Study findings related to the association between the suicide syndemic and HIV testing are particularly noteworthy. Individuals experiencing higher levels of suicidal behavior may be less likely to engage in HIV testing, potentially due to various psychosocial barriers, including structural stigma, discrimination, fear, and disengagement from mental health services [6]. Future studies should consider intervention approaches to promote engagement in the HIV prevention continuum, including HIV testing, among individuals who screen for suicidality.

Furthermore, the indirect pathways revealed in the analysis highlight the mediating roles of internalized homophobia and depression symptoms in the relationship between suicidal behavior and HIV testing. Internalized homophobia and depression symptoms not only directly impact suicidal behavior but also indirectly influence HIV testing behavior, underscoring the importance of addressing these underlying psychosocial factors in HIV prevention efforts targeting young BMSM. By acknowledging the interplay between suicide and HIV testing and implementing strategies that cater to the mental health needs of at-risk individuals, we can make strides in lowering barriers to HIV testing and enhancing the overall well-being of vulnerable populations.

Overall, these findings underscore the urgent need for comprehensive, community-centered, and competent interventions that address mental health and psychosocial well-being among young BMSM while also integrating HIV prevention strategies. Community-engaged frameworks like the Meaningful Involvement of People Living with HIV/AIDS Framework [81, 87] for sexual minority men of color can be adapted to inform the development of targeted HIV prevention strategies and services. Providing accessible and culturally competent mental health services for individuals at risk for suicide or living with HIV can help address underlying mental health challenges that may be impacting their willingness to engage in HIV testing. Working to reduce the stigma surrounding suicide, mental health, and HIV can create a more supportive environment that encourages individuals to seek help, including HIV testing and mental health services, without fear of judgment or discrimination. By addressing the intersecting challenges of internalized homophobia, depression, and suicidal behavior, tailored interventions have the potential to promote holistic health and well-being among young BMSM while improving outcomes in the HIV prevention continuum [6, 79, 80, 88].

## Limitations

While this study provides valuable insights into the complex interplay between suicidal behavior, internalized homophobia, depression symptoms, and HIV testing among young BMSM, several limitations should be acknowledged. First, given the limited survey data, the study did not examine potential protective factors, such as social support and networks, resilience, or coping strategies, which may mitigate the impact of risk factors and influence HIV testing behavior. Future research should consider incorporating protective factors to provide a more comprehensive understanding of the factors influencing HIV testing uptake among young BMSM. Second, the study utilized a cross-sectional design. Longitudinal studies are needed to assess temporal relationships and determine the causal pathways over time. Third, the data collected in this study relied on self-report measures, which are subject to recall bias and social desirability bias. Lastly, the Center for Epidemiological Studies—Depression has certain limitations. Cultural biases may influence how individuals respond to the CES-D, which can affect the results of international or multiethnic studies. Future research should consider incorporating biomarkers, collecting electronic medical records, and using other multimethod approaches to enhance the validity and reliability of the data. Finally, the study sample consisted of young BMSM recruited from specific geographic locations, which may limit the generalizability of the findings to other populations or settings.

## Conclusion

This study sheds light on the alarming rates of suicidal behavior among young BMSM, emphasizing the urgent need for targeted mental health interventions and HIV prevention services. These services must be tailored to address the unique psychosocial, social, and structural conditions affecting young BMSM, including racism, discrimination, and lack of access to mental health resources. Consistent with existing literature, our findings highlight the prevalence of internalized homophobia and depression symptoms among young BMSM, underscoring the detrimental potential impact of societal stigma and discrimination on their mental health.

The suicide syndemic framework offers a comprehensive approach to understanding the complex interplay of suicidal behaviors and HIV testing among young BMSM. Notably, our findings reveal a concerning association between the suicide syndemic and decreased engagement in HIV testing, suggesting the need for targeted interventions to promote HIV testing uptake. Moving forward, comprehensive community-centered interventions that address the intersecting challenges of mental health and HIV prevention are imperative to promote comprehensive health and well-being among young BMSM and improve outcomes within the HIV prevention continuum.

## Data Availability

The data underlying this article will be shared on reasonable request to the corresponding author.
